# Endothelin‐converting enzyme‐1c promotes stem cell traits and aggressiveness in colorectal cancer cells

**DOI:** 10.1002/1878-0261.12609

**Published:** 2019-12-19

**Authors:** Pablo Pérez‐Moreno, Sebastián Indo, Ignacio Niechi, Hernán Huerta, Pablo Cabello, Lilian Jara, Francisco Aguayo, Manuel Varas‐Godoy, Verónica A. Burzio, Julio C. Tapia

**Affiliations:** ^1^ Departamento de Oncología Básico Clínica Facultad de Medicina Universidad de Chile Santiago Chile; ^2^ Departamento de Tecnología Médica Facultad de Medicina Universidad de Chile Santiago Chile; ^3^ Instituto de Bioquímica y Microbiología Facultad de Ciencias Universidad Austral de Chile Valdivia Chile; ^4^ Programa de Genética Humana Instituto de Ciencias Biomédicas Facultad de Medicina Universidad de Chile Santiago Chile; ^5^ Centro de Investigación Biomédica Facultad de Medicina Universidad de Los Andes Santiago Chile; ^6^ Facultad de Ciencias de la Vida Universidad Andrés Bello Santiago Chile; ^7^ Fundación Ciencia & Vida Andes Biotechnologies SpA Santiago Chile; ^8^ Present address: Programa de Virología Instituto de Ciencias Biomédicas Facultad de Medicina Universidad de Chile Santiago Chile; ^9^ Present address: Centro de Biología Celular y Biomedicina Facultad de Medicina y Ciencia Universidad San Sebastián Santiago Chile

**Keywords:** CK2, phosphorylation, endothelin‐1, endothelin‐converting enzyme, cancer stem cell, aggressiveness

## Abstract

Endothelin‐1 is a mitogenic peptide that activates several proliferation, survival, and invasiveness pathways. The effects of endothelin‐1 rely on its activation by endothelin‐converting enzyme‐1 (ECE1), which is expressed as four isoforms with different cytoplasmic N termini. Recently, isoform ECE1c has been suggested to have a role in cancer aggressiveness. The N terminus of ECE1c is phosphorylated by protein kinase CK2 (also known as casein kinase 2), and this enhances its stability and promotes invasiveness in colorectal cancer cells. However, it is not known how phosphorylation improves stability and why this is correlated with increased aggressiveness. We hypothesized that CK2 phosphorylation protects ECE1c from N‐terminal ubiquitination and, consequently, from proteasomal degradation. Here, we show that lysine 6 is the *bona fide* residue involved in ubiquitination of ECE1c and its mutation to arginine (ECE1c^K6R^) significantly impairs proteasomal degradation, thereby augmenting ECE1c stability, even in the presence of the CK2 inhibitor silmitasertib. Furthermore, colorectal cancer cells overexpressing ECE1c^K6R^ displayed enhanced cancer stem cell (CSC) traits, including increased stemness gene expression, chemoresistance, self‐renewal, and colony formation and spheroid formation *in vitro*, as well as enhanced tumor growth and metastasis *in vivo*. These findings suggest that CK2‐dependent phosphorylation enhances ECE1c stability, promoting an increase in CSC‐like traits. Therefore, phospho‐ECE1c may be a biomarker of poor prognosis and a potential therapeutic target for colorectal cancer.

## Graphical abstract

CK2 phosphorylates the N terminus of ECE1c, impairing its proteasome‐dependent degradation and promoting invasiveness in colorectal cancer cells. We show that lys‐6 is the ubiquitinated residue and its mutation to arginine confers super‐stability to ECE1c. Moreover, overexpressed ECE1c^K6R^ mutant significantly promotes cancer stem cell‐like traits and hence higher aggressiveness. In conclusion, CK2 inhibition may have a promising therapeutic use in colorectal cancer.

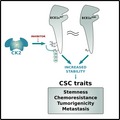

**Abbreviations**
CRC: colorectal cancer
CSC: cancer stem cell
ECE1c: endothelin‐converting enzyme isoform 1c
EMT: epithelial–mesenchymal transition
ET‐1: endothelin‐1

## 1 Introduction

Colorectal cancer (CRC) is the third‐leading cause of cancer‐related deaths in developed countries. Nearly half of CRC patients die from liver, lung, or brain metastases (Brenner *et al.*, 2014; Marley and Nan, 2016). Thus, there is a need for greater knowledge on the molecular and cellular bases of the aggressiveness observed in the evolution of CRC. Recent research has identified a new group of cells known as cancer stem cells (CSCs). These cells represent only 0.1–1% of the tumor mass but may be responsible for the genesis, recurrence, and metastasis of cancers. CSCs express a variety of genes associated with stemness, such as *Nanog*, *c‐Myc,* and *Stat‐3*, as well as surface markers such as LGR5, CD44, and CD133 (Fanali *et al.*, 2014; Hadjimichael *et al.*, 2015), which are being used to detect these cells. CSCs are also characterized by high resistance to antineoplastic drugs (Kreso and Dick, 2014; Rao *et al.*, 2013), likely attributable to expression of ATP‐binding cassette (ABC) transporter genes such as ABCB1 and ABCG2, which expel the drugs from the cancer cells, conferring chemoresistance and promoting tumor recurrence in chemotherapy‐treated CRC patients (Begicevic and Falasca, 2017; Fanali *et al.*, 2014). Interestingly, a transcriptomic analysis performed with putative CSCs isolated from CD133^+^ CRC cells revealed that the endothelin‐1 gene (END‐1) is also highly expressed in these cells (Puglisi *et al.*, 2011).

Endothelin‐1 (ET‐1) is a 23‐residue peptide and a member of the ET‐1 axis. ET‐1 activates various signaling pathways through the cancer‐related receptor, ET_A_R. These pathways include MAPK, PI3K/Akt, and Wnt/β‐catenin, which are important for cell proliferation, survival, and stemness phenotype (Puglisi *et al.*, 2011; Rosanò *et al.*, 2013, 2014). Some studies have shown that aberrant activation of Wnt/β‐catenin in LGR5^+^/PROM1^+^/BMI‐1^+^ CSCs is important for CRC tumorigenesis and tumor progression in murine models (Barker *et al.*, 2009; Sangiorgi and Capecchi, 2008; Yanai *et al.*, 2017; Zhu *et al.*, 2009). Indeed, CSC‐dependent CRC metastasis has been demonstrated in animal models, where LGR5^+^ expression has been found to be critical for the formation and maintenance of liver colonies (de Sousa e Melo *et al.*, 2017). In spite of the robust effects of ET‐1 on tumor malignancy, its half‐life is 1–2 min, and therefore, its biological effects are totally dependent on the maintenance of a critical concentration brought about by conversion of its precursor, big‐ET‐1, to ET‐1 by the endothelin‐converting enzyme‐1 (ECE1).

There are four isoforms of ECE1 (a, b, c, and d), which have identical transmembrane and extracellular C‐terminal domains but differ at the cytoplasmic N terminus. Each isoform displays a distinct subcellular localization and, in some cases, effect on cancer‐related properties such as proliferation and invasiveness (Tapia and Niechi, 2019). The N‐terminal end of isoform ECE1c is extraordinarily conserved between different animal species, showing complete identity in some residues. ECE1c is phosphorylated by protein kinase CK2 (also known as casein kinase 2), which enhances its stability. Moreover, overexpression of a phospho‐mimetic mutant promoted migration and invasiveness of DLD‐1 colon cancer cells (Niechi *et al.*, 2015). This evidence and a few more in the literature has let us recently to suggest that ECE1c contributes to cancer aggressiveness and may have a role as a regulator of cancer progression (Tapia and Niechi, 2019). Nonetheless, the mechanism by which CK2 phosphorylation enhances the stability of ECE1c remains to be unveiled.

CK2 was identified as a metastasis‐associated gene in a proteomic study using several CRC cell lines (Liang *et al.*, 2007)**,** and high CK2 levels correlated with poor patient prognosis (Lin *et al.*, 2011). Moreover, CK2 is elevated in a wide variety of cancers and is associated with increased growth and proliferation (Ruzzene and Pinna, 2010). Very recently, inhibition of CK2 with silmitasertib was shown to promote early methuosis‐like cell death in CRC cells, decreasing tumorigenicity at advanced treatment times (Silva‐Pavez *et al.*, 2019). CK2 promotes CRC cell survival through activation of the canonical Wnt pathway (Ponce *et al.*, 2011), which increases the expression of many β‐catenin targets, including both the inhibitor of apoptosis survivin and cyclooxygenase‐2 (Tapia *et al.*, 2006; Yefi *et al.*, 2011). In fact, ET‐1 itself is another β‐catenin target and has been found to be elevated in several cancers (Puglisi *et al.*, 2011; Rosanò *et al.*, 2013, 2014).

Protein degradation is often driven by the ubiquitin‐proteasome system, and lysine ubiquitination is the best‐known promoting signal in this system (Clague and Urbé, 2010). Interestingly, an *in silico* comparative analysis of the ECE1c amino acid sequences of several species performed by our group showed a conserved lysine at position 6, which is located near the CK2 phosphorylated serines 18 and 20 at the N terminus of ECE1c (P. Pérez‐Moreno, C. Quezada‐Meza, C. Chavez‐Almarza, E. Silva‐Pavez, F. Aguayo, I. Niechi, L. Jara, V. A.Burzio, A. Cáceres‐Verschae, M. Varas‐Godoy, V. M. Díaz, A. García de Herreros, & J. C. Tapia, unpublished data). Nevertheless, the potential role for Lys‐6 in promoting the stability of ECE1c or the stemness traits observed in colorectal cancer cells remains unexplored. In this work, we demonstrate that Lys‐6 is indeed crucial for the stability of ECE1c and that its mutation to arginine significantly increases the stability of this protein, even in the presence of the specific CK2 inhibitor silmitasertib. Moreover, colorectal cancer cells that overexpressed a super‐stable ECE1c mutant displayed traits characteristic of CSCs *in vitro* and *in vivo*. These findings strongly suggest that a CK2‐dependent increase in ECE1c stability promotes the generation of aggressive stem‐like CRC cells.

## 2 Materials and methods

### 2.1 Cell culture

DLD‐1 colorectal cancer cells (ATCC CCL‐221, Manassas, VA, USA) were provided by J. Silvio Gutkind (Department of Pharmacology, UC San Diego Moores Cancer Center). HT‐29 (ATCC HTB‐38) and SW‐480 (ATCC CCL‐228) colorectal cancer cells were donated by A. Quest (Universidad de Chile). Nontumor CHO‐K1 cells (ATCC CCL‐61) were a kind donation from M. Molina (Universidad de Chile). Cells were cultured in RPMI‐1640 medium (Invitrogen, Paisley, UK) supplemented with 10% fetal bovine serum (HyClone, Logan, UT, USA) and antibiotics (10 000 U·mL^−1^ penicillin and 10 μg·mL^−1^ streptomycin) in a humidified culture chamber at 37 °C, under a 5% CO_2_ atmosphere. All experiments for this study were performed within 1 year upon thawing, and cells were discarded after a maximum of 15 passages. Mycoplasma contamination was tested monthly using the EZ‐PCR Mycoplasma Test kit (Biological Industries, Beit Haemek, Israel).

### 2.2 Lentiviral cell cloning

Full‐length wild‐type ECE1c cDNA with in‐frame 5′‐Flag and Myc tags was inserted into the multicloning region of plasmid pLVX‐IRES‐mCherry (Clontech, Mountain View, CA, USA). Site‐directed mutagenesis was performed using the GENEART kit (Invitrogen) according to manufacturer’s instructions. Lenti‐X 293T cells were transfected with 8 μg psPax2 (encoding Gag‐Pol protein), 8 μg pLVX‐IRES‐mCherry (encoding ECE1c^WT^, ECE1c^K6R^ or empty), and 4 μg pCMV‐VSVg (encoding VSV G‐glycoprotein) and then suspended in 500 μL 0.25 m CaCl_2_. At 48 h post‐transfection, supernatants containing pseudotyped particles were harvested and passed through a cellulose acetate filter with a pore size of 0.45 μm. Viral particles were purified and concentrated by ultracentrifugation at 150 000 ***g*** for 75 min in SureSpin 630 rotor (Thermo Fisher, Vilnius, Lithuania) through a 25% sucrose cushion (TNE‐Sucrose 25%). Finally, cells were cultured at 5 × 10^4^ cells/well in 12‐well plates along with the recombinant lentiviruses at a MOI of 5 under normal growth conditions. Expression of mCherry was examined 72 h post‐transduction under a Nikon Eclipse TS100 Inverted Microscope (Nikon, Tokyo, Japan) equipped with epifluorescence. Cells were expanded for 1 week, and the brightest (mCherry^+^) cells were sorted on a FACSAria Fusion cell sorter (Becton‐Dickinson, San Jose, CA, USA).

### 2.3 Flow cytometry

For CD133^+^/CD44^+^ population analysis, 1 × 10^5^ cells were incubated with 5 μL (0.25 μg) 7‐AAD (BioLegend) as a viability marker and then with anti‐CD133/APC and anti‐CD44/BV‐421 antibodies (BioLegend, San Diego, CA, USA; 1 μL/1 × 10^5^ cells, diluted in 200 μL PBS/2% FBS) for 30 min. Unlabeled cells, APC mouse IgG1ƙ and BV‐421 mouse IgG1ƙ isotypes (BioLegend) were used as controls. For side population assay, cells were treated with 200 μm verapamil (Sigma‐Aldrich, St. Louis, MO, USA), incubated with Vibrant DyeCycle violet Stain (Invitrogen), and finally washed and prepared for analysis in a Becton‐Dickinson LSRFortessa X‐20 flow cytometer. Analyses were performed using facsdiva 8.02 software (San Jose, CA, USA) at the MED.UCHILE‐FACS Facility (Facultad de Medicina, Universidad de Chile).

### 2.4 Western blot

Cells were washed in ice‐cold PBS and sedimented at 1000 ***g*** for 10 min at RT. Pellets were suspended in RIPA buffer (10 mm Tris/HCl, pH 7.4, 1% sodium deoxycholate, 1% Triton X‐100, 0.1% SDS) containing 1 mm PMSF and protease inhibitor cocktail (Sigma‐Aldrich). Protein concentration was quantified using Bicinchoninic acid (Thermo Scientific, Rockford, IL, USA). Total proteins were separated by SDS/PAGE and transferred to Porablot NCP membranes (Macherey‐Nagel, Düren, Germany). Blots were probed with anti‐FLAG (1 : 2000; Sigma‐Aldrich), anti‐E‐cadherin (1 : 2000; Cell Signaling Technology, Danvers, MA, USA), anti‐N‐cadherin (1 : 2000; Cell Signaling Technology), anti‐Snail (1 : 2000; Cell Signaling Technology), and β‐actin (1 : 2000; Santa Cruz Biotechnology, Dallas, TX, USA) antibodies. Primary antibody binding was detected with anti‐goat IgG‐HRP (1 : 2000; Santa Cruz Biotechnology), anti‐mouse IgG‐HRP (1 : 2000; Santa Cruz Biotechnology), or anti‐rabbit IgG‐HRP (1 : 2000; Santa Cruz Biotechnology). Membranes were revealed using the EZ‐ECL chemiluminescence kit (Biological Industries, Haemek, Israel) and the ChemiDoc Touch Gel Imaging System (Bio‐Rad, Hércules, CA, USA).

### 2.5 Protein stability

Cells (5 × 10^5^) were seeded into P60 plates and cultured for 36 h in complete medium under normal conditions, with 20 μg·mL^−1^ cycloheximide (CHX) in the absence or presence of 25 μm silmitasertib (ApexBio Technology LLC, Houston, TX, USA). At different time points, cells were harvested and lysed and 30 μg of total protein was separated by SDS/PAGE. ECE1c was detected by western blot using a mouse anti‐FLAG antibody (1 : 2000; Sigma‐Aldrich).

### 2.6 RT‐qPCR

Total RNA was extracted from cells using the EZNA Total RNA Kit I (Omega Bio‐tek, Norcross, GA, USA) and treated with DNase (Ambion DNA‐free kit; Life Technologies, Carlsbad, CA, USA). RNA concentration was measured using a NanoQuant Infinite M200 pro spectrophotometer (Tecan, Männedorf, Zurich, Switzerland). Reverse transcription was performed with total RNA, using the AffinityScript QPCR cDNA Synthesis Kit (Agilent Technologies, Bastrop, TX, USA), according to manufacturer’s directions. Quantitative real‐time PCR was performed in a StepOne real‐time PCR system (Applied Biosystems) with SYBR Green PCR Master Mix (Thermo Fisher Scientific). The PCR protocol was as follows: a first step at 95 °C for 30 s, followed by 40 cycles of 95 °C for 5 s and 60 °C for 30 s, finishing with a cycle of 95 °C for 15 s, 60 °C for 1 min, and 95 °C for 15 s. Reactions were performed in triplicate, and the relative abundance of each mRNA was determined by using the $2^{-\Delta \Delta C_{t}}$ method and normalized to GAPDH. The 95% confidence interval was determined to indicate the variability of the mean ratios for each experiment. Primers used were the following: Nanog Fw 5′‐AACCAAAGGATGAAGTGCAAGCGG‐3′, Rv 5′‐TCCAAGTTGGGTTGGTCCAAGTCT‐3′; Lgr5 Fw 5′‐GATGTTGCTCAGGGTGGACT‐3′, Rv 5′‐GGGAGCAGCTGACTGATGTT‐3′; Stat3 Fw 5′‐CACCGAGTCGTAGTCGAGGT‐3′, Rv 5′‐CCTCTGCCGGAGAAACAG‐3′; c‐Myc Fw 5′‐CTGCTCCAGGTACCGTGTGT‐3′, Rv 5′‐TTTGGGGTAGTGGAAAACCA‐3′; CD133 Fw 5′‐TTTTGGATTCATATGCCTTCTGT‐3′, Rv 5′‐ACCCATTGGCATTCTCTTTG‐3′; CD44 Fw 5′‐CGTGGAATACACCTGCAAAG‐3′, Rv 5′‐CGGACACCATGGACAAGTTT‐3′ and GAPDH Fw 5′‐GAGTCAACGGATTTGGTCGT; Rv 5′‐GACAAGCTTCCCGTTCTCAG‐3′.

### 2.7 Sphere formation

Cells (5 × 10^4^) were seeded into ultra‐low attachment 6‐well plates (Corning, Life Sciences, Corning, NY, USA) and cultured under normal conditions for 7 days in mammary epithelial cell growth media (MEGM) supplemented with 25 ng·mL^−1^ EGF, 0.5 g·mL^−1^ hydrocortisone, 5 μg·mL^−1^ insulin (Lonza, Basel, Switzerland), and 25 ng·mL^−1^ b‐FGF (Invitrogen). Spheres> 100 μm in diameter were counted and measured. Quantification was carried out with micrometrics se premium 4 software (Accu‐Scope, Commack, NY, USA).

### 2.8 Colony formation

Cells (2.5 × 10^3^) were suspended in 0.33% Bacto agar (BD Biosciences, Heidelberg, Germany) in RPMI‐1640 containing 12.5% FBS. The cell suspension was then poured into 6‐well plates containing a layer of 2 mL 0.5% agar. Plates were fed twice a week with 0.2 mL RPMI‐1640 supplemented with 10% FBS. After 21 days, colonies were photographed under a Nikon Eclipse TS100 Inverted Microscope. Finally, colonies were stained using 0.005% crystal violet dissolved in 20% methanol for 30 min at RT, photographed with a Nikon D5100 camera, and counted using imagej software (NIH, Bethesda, MD, USA).

### 2.9 Cell invasion

Cells (4 × 10^4^) growing in FBS‐free medium were seeded in Matrigel invasion chambers (Corning) and placed on 24‐well plates with 500 μL RPMI/10% FBS per well. Cells were incubated for 22 h at 37 °C and 5% CO_2._ Matrigel chambers were stained and fixed with 0.05% crystal violet in 20% methanol for 1 h. Invasive cells were counted under a Nikon Eclipse TS100 Inverted Microscope.

### 2.10 *In vivo* assays

All experimental protocols and mice care were performed in accordance with institutional guidelines and approved by the local Bioethical Committee (protocol CBA‐436 FMUCH, Facultad de Medicina, Universidad de Chile), as well as performed in accordance with worldwide guidelines published elsewhere (Morton and Griffiths, 1985). Female 6‐week‐old BALB/c NOD/SCID mice weighing 14–16 g were randomly divided into three cohorts of three animals each. For tumorigenesis experiments, 2 × 10^6^ DLD‐1 cells transduced with lentiviral constructs, in 100 μL saline (0.9% NaCl), were injected subcutaneously into each mouse. Tumor length and width was measured every 4 days. Relative volume was calculated with the formula π/6 × *L* × *W* ^2^ as described elsewhere (Tomayko and Reynolds, 1989). Mice were euthanized on day 22, and tumors were extracted, photographed, fixed in 4% formaldehyde, and stored in paraffin for histological analysis. For metastasis experiments, mice were divided into three cohorts of six animals each. Cells (2 × 10^6^) were suspended in 100 μL 0.9% NaCl and injected into the lateral tail vein. Mice were monitored and euthanized at 75 days or earlier if suffering was evident. Lungs were removed, fixed in 4% formaldehyde, and finally stored in paraffin for histological analysis. G*Power 3 was used to calculate the minimal number of animals to test the hypothesis. A power of 90%, a significance level of 0.05 (two sided), and using a Wilcoxon test, as well as two‐ and threefold differences as significant primary outcomes were considered for tumorigenesis and metastasis, respectively, since these differences have been typically observed in our *in vitro* experiments.

### 2.11 Immunohistochemistry

Tissue sections (3 μm) were deparaffinized with xylene and rehydrated through a decreasing ethanol concentration series, finalizing in distilled water. Antigen retrieval was performed in 10 mm citrate buffer (pH 6.0) for 30 min at 92 °C. Nonspecific sites were blocked with horse serum for 30 min at RT. Samples were incubated with the primary antibodies anti‐Ki67 (1 : 50; Santa Cruz Biotechnology) or anti‐FLAG (1 : 100; Sigma‐Aldrich) overnight at 4 °C. After washing inPBS, primary antibodies were detected by incubation with a universal secondary antibody (R.T.U. Universal Secondary Antibody; Vector Laboratories, Burlingame, CA, USA) for 30 min at RT. Samples were washed and incubated with ABC reagent for 30 min, then revealed with DAB. FLAG‐stained samples were counterstained with Mayer’s hematoxylin. Results were documented under a Leica DM 2500 microscope provided with a Leica DFC 425C camera (Leica, Wetzlar, Germany).

### 2.12 Statistical analysis

Values were plotted as mean ± SEM from at least three independent experiments. Depending on the experiments, statistical differences were determined by using the graphpad prism5 software (San Diego, CA, USA) on the raw data with either ANOVA and Tukey post‐test, *t*‐test, or log‐rank test. A *P*‐value ≤ 0.05 was considered significant.

## 3 Results

### 3.1 Lysine 6‐to‐arginine change promotes super‐stability of ECE1c

**Figure 1:**
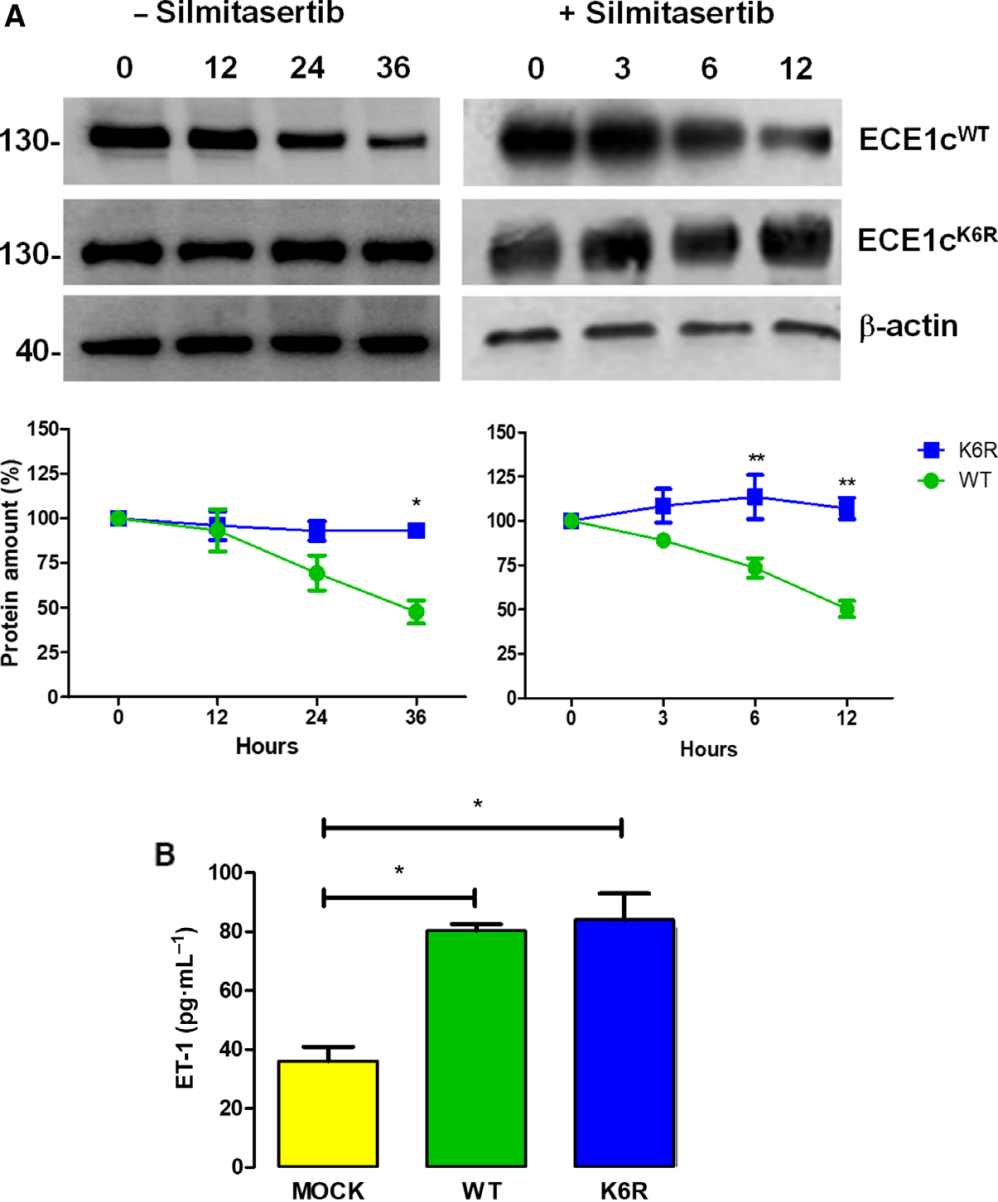
Mutation of Lys‐6 to Arg enhances stability of ECE1c. (A) DLD‐1 clone cells expressing Flag‐tagged ECE1c^WT^ or ECE1c^K6R^ proteins were incubated with 20 μg·mL^−1^ cycloheximide (CHX) in the absence (for 36 h) or presence (for 12 h) of 25 μm silmitasertib. ECE1c proteins were detected by western blot with an anti‐Flag antibody, using β‐actin as loading control. Representative blots are shown (upper). Relative levels (%) of Flag‐ECE1c proteins from three independent experiments were calculated (lower). (B) DLD‐1 clones described in A were grown in medium without FBS for 48 h. Culture supernatants were used to measure ET‐1 levels using ELISA according to manufacturer’s instructions (Thermo Fisher). Data represent average ± SEM (*n* = 3). ANOVA and Tukey tests were used. *P ≤ 0.05, **P ≤ 0.01.

The N terminus of ECE1c has been shown to be phosphorylated by protein kinase CK2 at residues Ser‐18 and Ser‐20, promoting stability, migration, and invasiveness in DLD‐1 colon cancer cells upon overexpression (Niechi *et al.*, 2015). An *in silico* analysis performed by our group, comparing the ECE1c amino acid sequences of several species, showed that the conserved Lys‐6, which is near serines‐18/20 phosphorylated by CK2, is a putative site for ubiquitination and therefore a signal for proteasomal degradation (Fig. S1). Therefore, Lys‐6‐to‐Arg (K6R) site‐directed mutagenesis was performed in order to block degradation and to assess the role of this residue in ECE1c stability. Lentiviral vectors harboring FLAG‐tagged ECE1c^K6R^ and ECE1c^WT^ were constructed in a bicistronic unit along with the fluorescent protein mCherry and used to transduce DLD‐1 cells. Once isolated and expanded, clones were treated with cycloheximide (CHX) and the protein stability of ECE1c was analyzed in the absence and presence of the CK2 inhibitor silmitasertib at various time points by western blot. As expected, ECE1c^WT^ levels remained relatively unchanged at 12 h in the absence of silmitasertib, dropping to around 50% at 36 h. In the presence of the drug, however, the 50% decrease was attained already at 12 h of incubation, showing that the stability of ECE1c^WT^ depends on CK2 activity (Fig. 1A). In contrast, ECE1c^K6R^ levels remained constant even in the presence of the CK2 inhibitor, suggesting that elimination of the ubiquitinable Lys‐6 rendered the stability of the protein independent from CK2 phosphorylation (Fig. 1A). ECE1c protein stability was also studied in lentiviral‐transduced CHO‐K1 cells, which express negligible endogenous levels of any ECE1 isoform (Niechi *et al.*, 2015). Results were essentially the same as those for DLD‐1 cells (data not shown), indicating that Lys‐6 is crucial for promoting stability of ECE1c in both nontumor and colorectal cancer cells. Notably, secreted levels of the ET‐1 peptide were elevated in both ECE1c^K6R^ and ECE1c^WT^‐expressing cells and were quite similar after 48 h of growth (Fig. 1B). Taken together, these data indicate that Lys‐6‐to‐Arg mutagenesis confers super‐stability to ECE1c^K6R^, although this change does not affect its catalytic production of ET‐1, which was indistinguishable between ECE1c^WT^‐ and ECE1c^K6R^‐expressing cells.

### 3.2 ECE1c^K6R^ induces expression of stemness genes and spheroidogenesis in CRC cells

**Figure 2:**
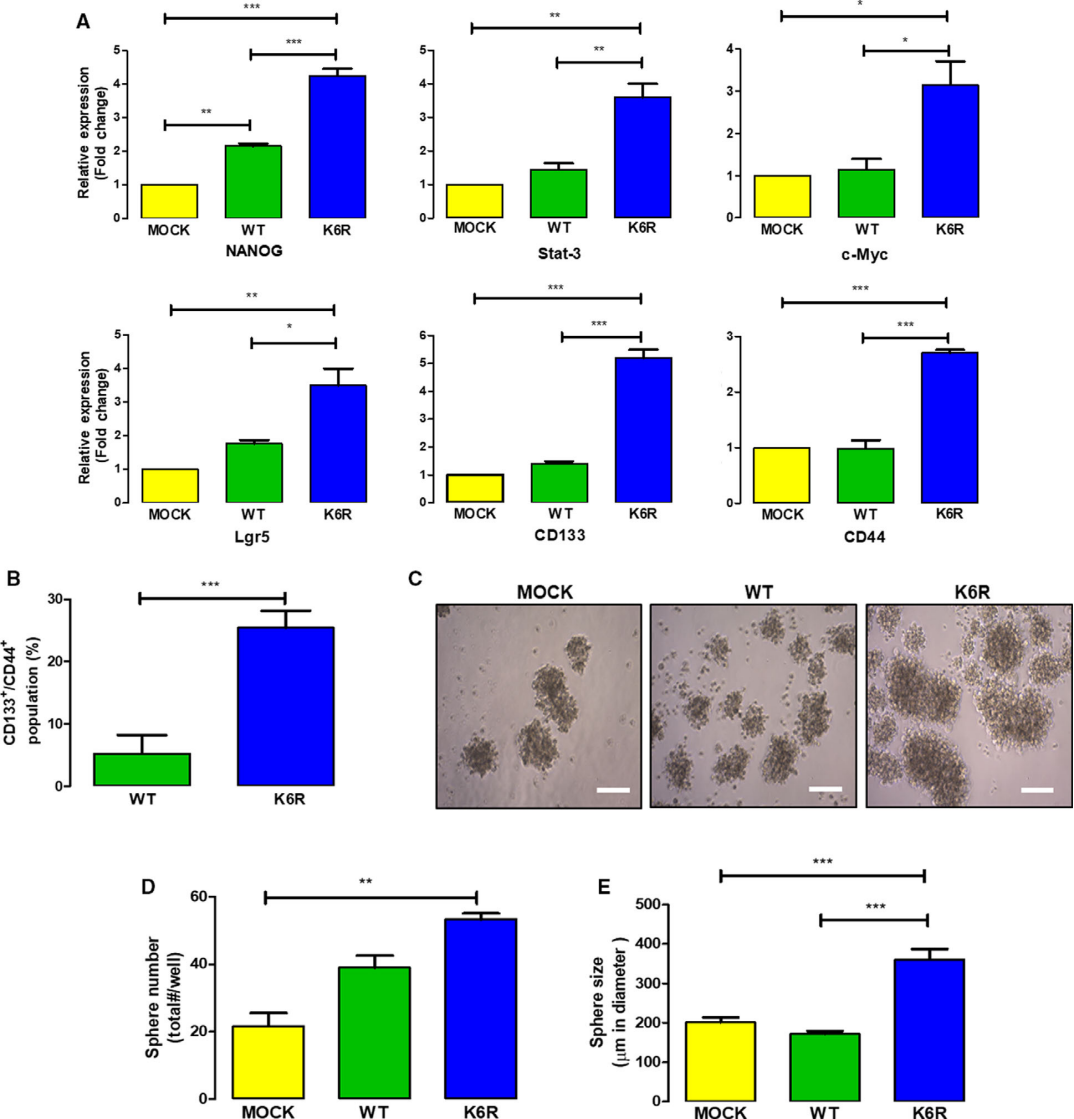
ECE1c^K6R^ expression induces stemness and spheroidogenesis. (A) DLD‐1 cells expressing either Flag‐tagged ECE1c^WT^ or ECE1c^K6R^ proteins were grown under normal conditions for 48 h, and the mRNA levels of stemness genes were quantified using RT‐qPCR (*n* = 3). (B) Cells grown as in A were evaluated for CD133^+^/CD44^+^ population using flow cytometry and flowjo V.10 software (*n* = 3). (C) As in A, cells were grown for 7 days under spheroidogenic conditions. Representative images are shown (scale bar = 150 µm). (D) Number of spheroids formed in C was determined from a triplicate analysis. (E) Sizes of spheroids formed in C were quantified from a triplicate analysis. Data represent average ± SEM (*n* = 3). ANOVA and Tukey tests were used, except for B: *t*‐test. *P ≤ 0.05, **P ≤ 0.01, ***P ≤ 0.001.

The ET‐1 axis is known to function through various signaling pathways that promote expression of genes linked to stemness (Rosanò *et al.*, 2013). Thus, we assessed whether our various DLD‐1 cell clones overexpressing either super‐stable ECE1c^K6R^ or ECE1c^WT^ expressed differential levels of mRNA for genes traditionally associated with stemness, such as Nanog, Stat‐3, c‐Myc, and Lgr5, as well as genes coding for stem cell surface markers, such as CD133 and CD44. Unexpectedly, all genes evaluated were significantly elevated in ECE1c^K6R^‐expressing cells as compared to mock cells (Fig. 2A). Moreover, twofold (Nanog and Lgr5) to fivefold (CD133) higher mRNA levels were observed in ECE1c^K6R^‐expressing cells as compared to ECE1c^WT^ cells. In addition, similar increases in mRNA levels of stemness genes were detected in equivalent clones from other CRC cells of different malignancy (Ahmed *et al.*, 2013), such as HT‐29 (Dukes C/D) (Fig. S2A) and SW‐480 (Dukes B) (Fig. S2B), which are positive and negative, respectively, for activating mutations in the *PIK3CA* gene, a common genetic trait found in CRC tumors. Moreover, the double‐positive CD133^+^/CD44^+^ population detected by flow cytometry in ECE1c^K6R^‐expressing cells was around 25%, while this population was only 5% in ECE1c^WT^‐expressing cells, correlating with the changes in the mRNA levels of these surface stemness markers (Fig. 2A). These results suggest that super‐stable ECE1c expression in DLD‐1 cells may enhance generation of CSCs *in vitro*, which likely have the ability to self‐renew and to form spheres growing in suspension. Thus, a spheroidogenesis assay was performed with the DLD‐1 cell clones for a period of 7 days (Fig. 2C). As expected, the number of spheres was higher than mock cells for both ECE1c^WT^‐ and ECE1c^K6R^‐expressing cells, although the latter was slightly higher (Fig. 2D). In addition, the spheres were significantly larger for ECE1c^K6R^‐expressing cells (Fig. 2E). Taken together, these results suggest that super‐stable ECE1c expression in CRC cells generates a subpopulation with a dramatically elevated expression of stemness genes and enhanced capacity to form self‐renewing spheres, which strongly suggests a CSC phenotype.

### 3.3 Enhanced drug resistance of stem‐like ECE1c^K6R^‐expressing CRC cells

**Figure 3:**
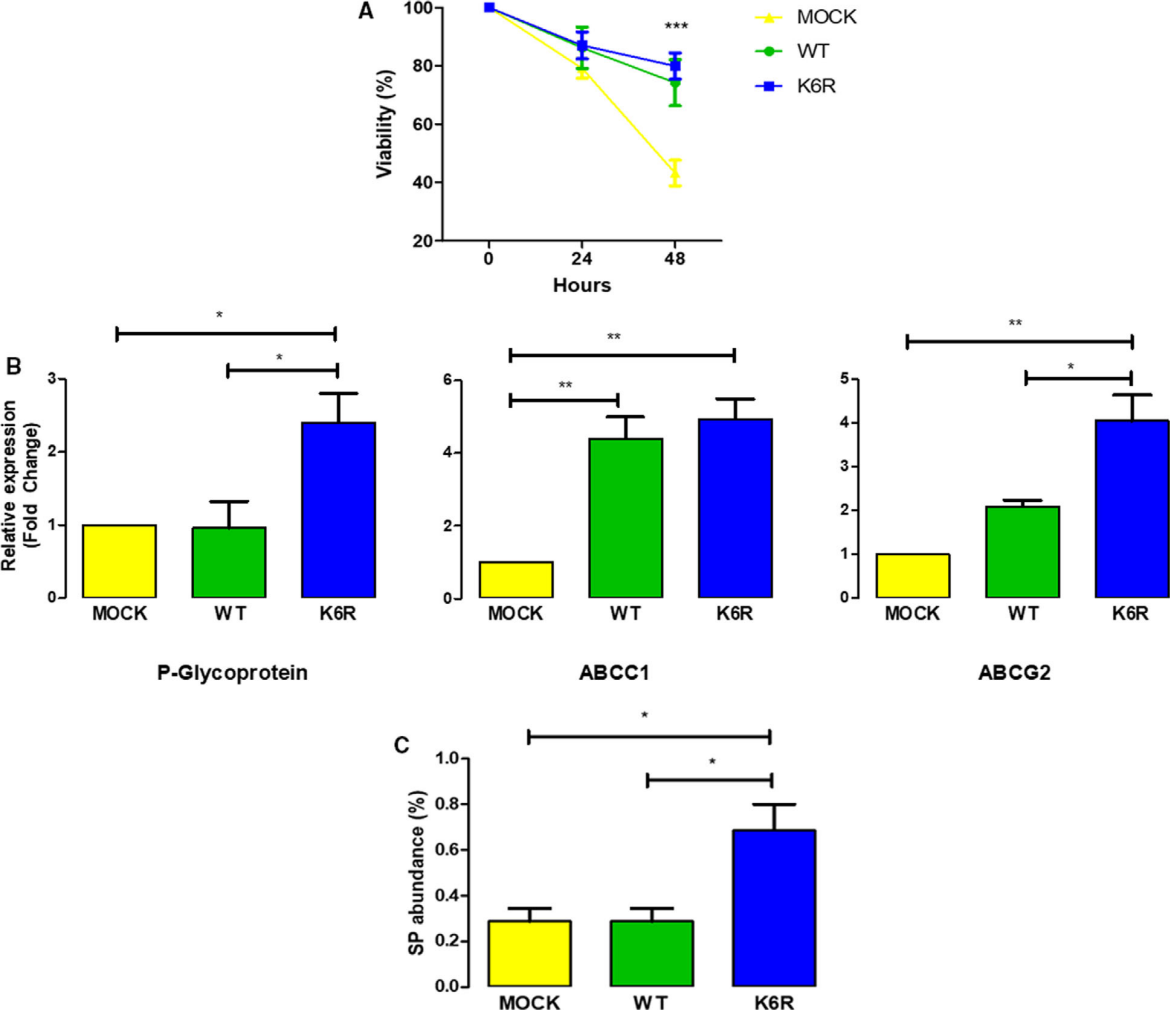
Stem‐like ECE1c^K6R^ cells display enhanced drug resistance. (A) DLD‐1 cells expressing either Flag‐tagged ECE1c^WT^ or ECE1c^K6R^ proteins (or Mock cells) were analyzed for cell viability using MTS assay following incubation with 25 μm 5‐FU for 48 h. (B) As in A, 10^6^ cells were grown under normal conditions for 48 h and further analyzed for mRNA levels of P‐Glycoprotein, ABCC1, and ABCG2 by RT‐qPCR. (C) As in A, 10^6^ cells were incubated in the presence or absence of 200 μm verapamil for 30 min and incubated with 5 μL DCV for another 30 min. Side population abundance was determined by flow cytometry. Data represent average ± SEM (*n* = 3). ANOVA and Tukey tests were used. *P ≤ 0.05, **P ≤ 0.01, ***P ≤ 0.001.

Chemoresistance is another CSC trait, which explains the recurrence of cancer in drug‐treated patients (Kozovska *et al.*, 2014). In order to explore the role of ECE1c in chemoresistance, we treated our cancer stem‐like ECE1c‐expressing (i.e., CS^ECE1c^) DLD‐1 clones with 5‐Fluorouracil (5‐FU), which is the main antineoplastic drug used in CRC. An MTS assay in the presence of 5‐FU showed that both CS^K6R^ and CS^WT^ cells were equally resistant to 5‐FU as compared to mock cells after 48 h of incubation (Fig. 3A). Elevated ABC pump gene expression has been associated with drug resistance, attributable to the efflux of drugs from the cells (Begicevic and Falasca, 2017). Indeed, CS^K6R^ cells showed significantly increased mRNA levels of both P‐Glycoprotein and ABCG2 in comparison with CS^WT^ cells after 24 h of growth. ABCC1 mRNA levels were also elevated in CS^K6R^ cells, although quite similarly compared to CS^WT^ cells (Fig. 3B). Finally, in order to correlate these results with a functional readout, a side population (SP) assay was performed. Efflux of DyeCycle Violet (DCV) in the presence or absence or presence of the pump inhibitor, verapamil, was evaluated with cytometry. As shown in Fig. 3C, while mock and CS^WT^ cells showed a similar abundance of SP cells, CS^K6R^ cells displayed a twofold higher abundance of SP cells that pumped out DCV. These results strongly suggest that expression of the super‐stable ECE1c^K6R^ dramatically enhances chemoresistance of stem‐like CRC cells.

### 3.4 Super‐stable ECE1c^K6R^ enhances anchorage‐independent growth and tumorigenesis

**Figure 4:**
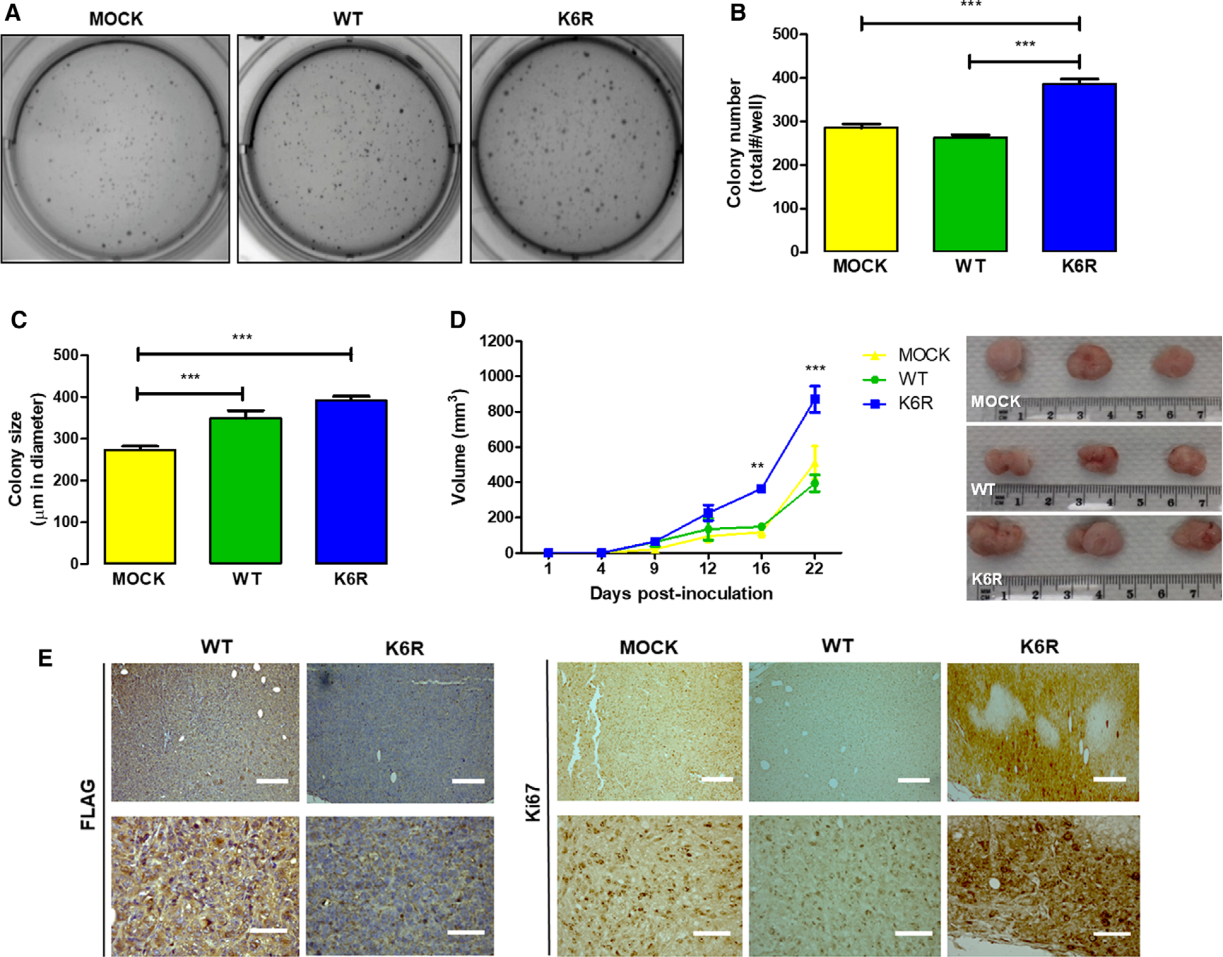
Increased tumor growth of stem‐like ECE1c^K6R^ cells *in vivo*. (A) DLD‐1 clone cells expressing either Flag‐tagged ECE1c^WT^ or ECE1c^K6R^ proteins (or Mock cells) were grown for 21 days in soft agar, and anchorage‐independent growth was determined. Representative images of crystal violet‐stained colonies obtained at a magnification of 40×. (B) Colonies formed from cells in A were counted and plotted as total colonies/well. (C) Sizes of colonies (μm in diameter) formed in A were measured using micrometrics se premium 4 software. (D) Cells (2 × 10^6^) were subcutaneously injected into NOD/SCID mice, and tumor volume (mm^3^) was monitored. After the mice were euthanized on day 22, tumors were photographed, measured, and fixed in paraffin. (E) Paraffin slices of tumors from the experiment in D were analyzed by IHC with anti‐FLAG (left) and anti‐Ki‐67 (right) antibodies (scale bars upper panels = 150 µm; lower panels = 50 µm). Data represent average ± SEM (*n* = 3). ANOVA and Tukey tests were used. **P ≤ 0.01, ***P ≤ 0.001.

Cancer stem cells are known to promote tumor growth, attributable to the capacity for self‐renewal of these cells (Kozovska *et al.*, 2014). This trait suggests that CS^K6R^ cells may show increased growth capacity in anchorage‐independent conditions such as those found in a soft agar assay, which is understood an *in vitro* surrogate for *in vivo* tumor growth. Thus, the number and size of colonies formed were determined in our putative CSC‐like cells after 21 days of growth in soft agar (Fig. 4A). Results showed that only CS^K6R^ cells showed a significantly greater number of large colonies as compared to mock and CS^WT^ cells (Fig. 4B,C). These results suggest that ECE1c^K6R^ also contributes to the ability of stem‐like CRC cells to drive tumor growth. To determine whether the super‐stability of ECE1c increases tumor growth *in vivo*, we used a xenograft model of immunocompromised NOD/SCID mice. We subcutaneously inoculated putative CSC‐like cells and measured tumor size over the following 22 days. As shown in Fig. 4D, CS^K6R^ cells enhanced tumor growth in mice as compared to mock and CS^WT^ cells. This difference was apparent from 12 days but became significantly greater after 16 days of inoculation. As expected from previous results, an IHC analysis showed that tumors formed from CS^K6R^ cells had significantly higher levels of Ki67, indicating increased proliferation (Fig. 4E). Taken together, these results demonstrate that enhanced stability of ECE1c exerts a significant effect on the tumor growth of putative CSC‐like cells *in vivo*.

### 3.5 Invasiveness and metastasis are enhanced in stem‐like ECE1c^K6R^ cells

**Figure 5:**
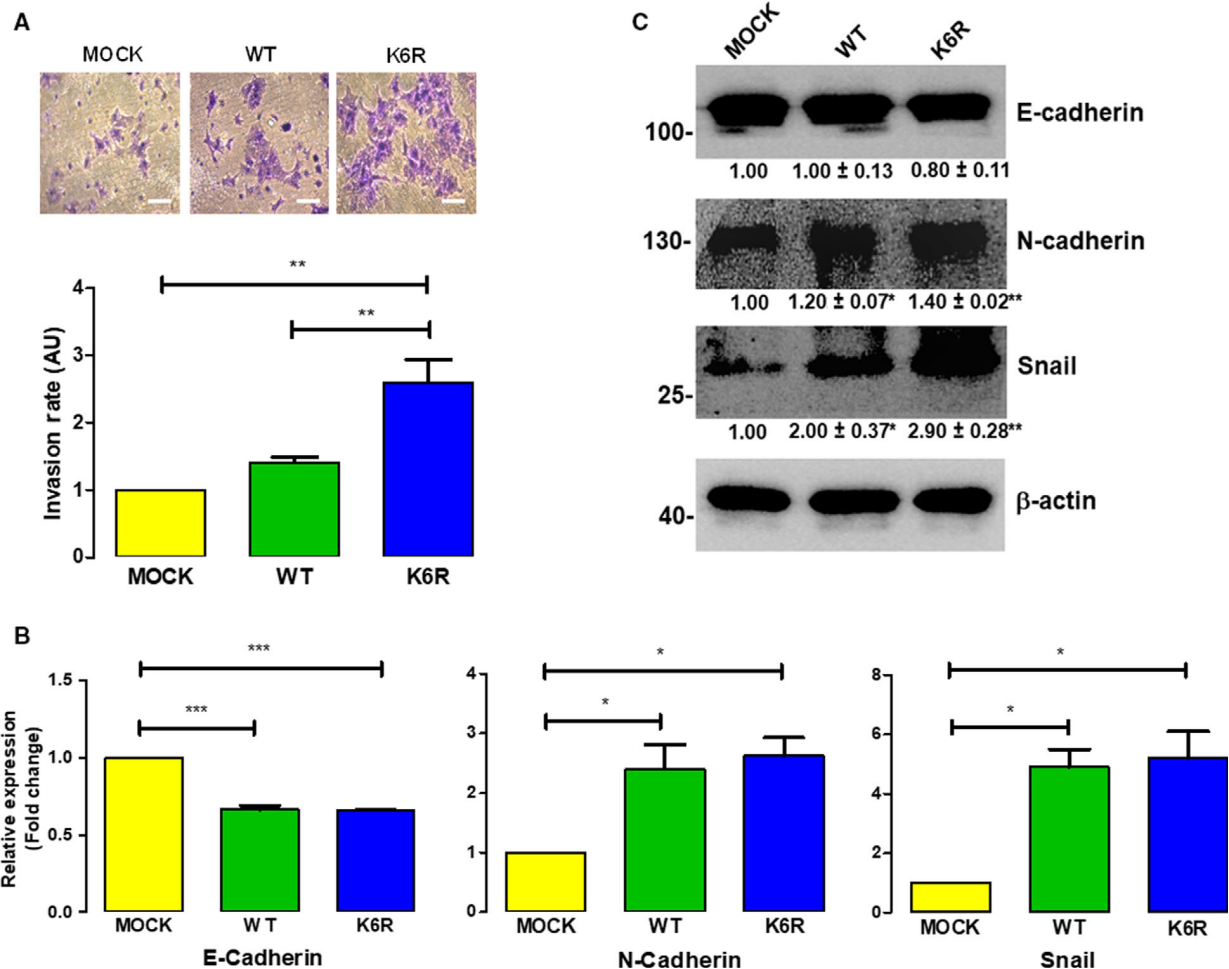
ECE1c^K6R^ expression promotes EMT‐linked invasiveness. (A) DLD‐1 clone cells expressing either Flag‐tagged ECE1c^WT^ or ECE1c^K6R^ proteins (or Mock cells) were grown for 22 h in matrigel chambers. Invasive capability was determined by counting crystal violet‐stained cells (upper panel: representative images; scale bar = 50 µm). Relative quantification of stained cells is shown (lower panel). (B) As in A, cells were grown under normal conditions for 48 h, and the mRNA levels of E‐cadherin, N‐cadherin, and Snail were determined using RT‐qPCR. (C) Protein levels of E‐cadherin, N‐cadherin, and Snail from cells in B were determined using western blot. Data represent average ± SEM (*n* = 3). ANOVA and Tukey tests were used. *P ≤ 0.05, **P ≤ 0.01, ***P ≤ 0.001.

**Figure 6:**
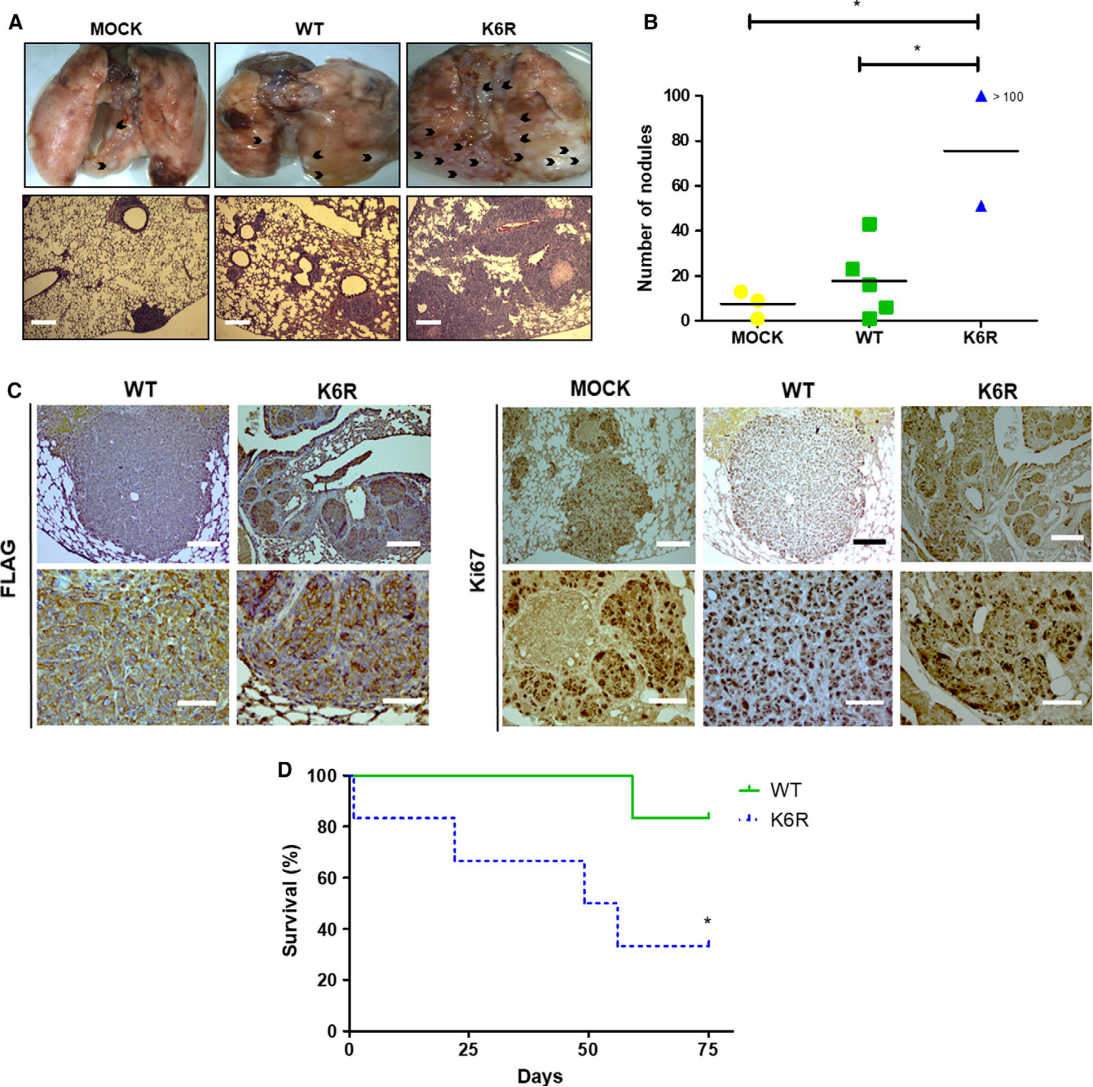
Stem‐like ECE1c^K6R^ cells display enhanced lung colonization capacity *in vivo.* (A) DLD‐1 cells expressing either Flag‐tagged ECE1c^WT^ or ECE1c^K6R^ proteins (2 × 10^6^) were intravenously injected into NOD/SCID mice. Animals were euthanized after 75 days of monitoring, or earlier if the animals showed signs of suffering. Representative images of metastatic lungs are shown, where black arrowheads indicate metastatic nodules (upper panel), along with HE staining of paraffin sections (lower panel; scale bar = 300 µm) (B) Numbers of metastatic nodules in each cohort from mice in A were determined from paraffin slices. (C) Paraffin slices of lungs from experiment in A were analyzed using IHC with anti‐FLAG (left) and anti‐Ki‐67 (right) antibodies (scale bars upper panels = 150 µm; lower panels = 50 µm). (D) Survival (%) of mice from experiment in A. Data represent average ± SEM (*n* = 3 each cohort). ANOVA and Tukey tests were used, except for B: log‐rank test. *P ≤ 0.05.

Another trait of CSCs is the ability to degrade the ECM in order to reach the bloodstream and produce distant metastases (de Sousa e Melo *et al.*, 2017). The invasive capacity of our stem‐like CRC clone cells was evaluated *in vitro* by matrigel assay. As expected, only CS^K6R^ cells showed significantly increased invasiveness (Fig. 5A), which was accompanied by increased N‐cadherin and Snail mRNA (Fig. 5B) and protein (Fig. 5C) levels, as well as decreased E‐cadherin mRNA levels, in accordance with events that are characteristic of epithelial–mesenchymal transition (EMT). Interestingly, despite their dramatically different invasive capacities that somehow coincided with significant mRNA levels, CS^K6R^ and CS^WT^ cells did not show important differences in E‐cadherin and N‐cadherin protein levels, suggesting that other complementary mechanism(s) are responsible for the augmented invasive capacity displayed by ECE1c^K6R^‐expressing DLD‐1 cells. Finally, to assess *in vivo* whether expression of the super‐stable ECE1c^K6R^ protein has a role in the metastatic capacity of our stem‐like CRC cells, we intravenously inoculated the cells into NOD/SCID mice and observed lung foci formation for up to 75 days (Fig. 6A). Results showed a significantly increased formation of lung nodes in mice inoculated with CS^K6R^ cells (Fig. 6B). An IHC analysis of lungs from mice inoculated with Flag‐expressing cells confirmed that large numbers of nodes were formed from CS^K6R^ cells and that the nodes were extremely proliferative according to Ki67 values (Fig. 6C). However, Ki67 levels in the CS^K6R^‐derived nodes were, surprisingly, indistinguishable from those of mock‐derived nodes, which suggests a similar proliferative potential for both cells in the lungs. This result was essentially contrary to that observed in the tumor growth assay, where CS^K6R^ cells displayed a significantly higher Ki67 index as compared to mock‐derived tumors (Fig. 4E). Interestingly, a pathological evaluation of samples indicated that all lung metastases presented traits of massive lymphangitic carcinomatosis, and the livers of some mice showed marked venous thrombosis. As expected from the above findings, the aggressiveness of the CS^K6R^ cells dramatically decreased survival in these mice (Fig 6D). In conclusion, these results demonstrate that super‐stable ECE1c^K6R^ expression in CRC cells promotes stemness‐related lung metastasis, which correlates with dramatically decreased survival *in vivo*.

## 4 Discussion

Most proteins are known to be ubiquitinated via a lysine residue for further degradation (Dikic, 2017; Swatek and Komander, 2016). Our findings indicate that Lys‐6‐to‐Arg mutation promotes a dramatic increase in ECE1c stability. Lysine 6 is located at the N terminus near serines 18 and 20, which we recently identified as substrates for CK2 phosphorylation in CRC cells (P. Pérez‐Moreno, C. Quezada‐Meza, C. Chavez‐Almarza, E. Silva‐Pavez, F. Aguayo, I. Niechi, L. Jara, V. A.Burzio, A. Cáceres‐Verschae, M. Varas‐Godoy, V. M. Díaz, A. García de Herreros, & J. C. Tapia, unpublished data). While the underlying mechanism is unclear, the enhanced stability of ECE1c could be a consequence of blocking ubiquitination and subsequent proteasomal degradation upon phosphorylation by CK2, since the mutation of Lys‐6 to Arg notably enhances the stability of the protein and this stability becomes independent of CK2 phosphorylation. Notably, CK2 is elevated in almost all types of cancers and is associated with increased malignancy and poor patient prognosis (Laramas *et al.*, 2007; Lin *et al.*, 2011; Ruzzene and Pinna, 2010). Importantly, CK2 regulates the stability of several proteins through phosphorylation. CK2 phosphorylates OTUB1 deubiquitinase, promoting its nuclear activity as well as the deubiquitination and stability of chromatin‐binding proteins (Herhaus *et al.*, 2015). CK2 also phosphorylates c‐Myc, which enhances transcription of genes involved in various hallmarks of cancer (Duncan and Litchfield, 2008). Moreover, metalloproteinases from the ECE1 family, such as angiotensin‐converting enzyme, have been observed to be ubiquitinated and degraded by the proteasome (Abdul‐Hafez *et al.*, 2017). We have previously reported that in the presence of the CK2 inhibitor silmitasertib, the proteasome inhibitor MG‐132 reverts degradation of a recombinant GST‐fused ECE1c N terminus (Niechi *et al.*, 2015). Additionally, our results with biphospho‐mimetic or biphospho‐resistant ECE1c mutants, where specific CK2‐phosphorylation of serines 18 and 20 enhances ECE1c stability (P. Pérez‐Moreno, C. Quezada‐Meza, C. Chavez‐Almarza, E. Silva‐Pavez, F. Aguayo, I. Niechi, L. Jara, V. A.Burzio, A. Cáceres‐Verschae, M. Varas‐Godoy, V. M. Díaz, A. García de Herreros, & J. C. Tapia, unpublished data), taken together with the above findings, strongly suggest that ECE1c degradation indeed occurs via the proteasome.

The expression of a subset of genes is associated with CSCs, including markers such as Lgr5, CD133, and CD44, as well as other genes involved in the acquisition of stemness traits in tumor cells, such as transcription factors Nanog, Stat‐3, and c‐Myc (Gong *et al.*, 2015; Hadjimichael *et al.*, 2015). In fact, these genes were significantly increased in our ECE1c^K6R^‐expressing DLD‐1 cells, which also showed increased acquisition of cellular properties typically associated with CSCs. Thus, our findings further suggest a role for the ET‐1 axis in tumorigenesis and a stem‐like phenotype (Cole *et al.*, 2008; Rennoll and Yochum, 2015; Rosanò *et al.*, 2013; Yan *et al.*, 2008, 2015; Yu *et al.*, 2017; Zeilstra *et al.*, 2008). In this axis, the mitogenic peptide ET‐1 binds to the cancer‐related receptor, ET_A_R, which cross‐activates various signaling pathways that lead to tumor development, including the Wnt/β‐catenin, Ras/MAPK, and PI3K/Akt pathways (Coffman *et al.*, 2013; Puglisi *et al.*, 2011; Rosanò *et al.*, 2013). Nevertheless, we did not address here which signaling pathway(s) participate in the acquisition of CSC‐like traits in CRC cells, and this issue warrants further research.

Equivalent production of the ET‐1 peptide was observed in ECE1c^WT^‐ and ECE1c^K6R^‐expressing clone cells. Although unexpected, this finding suggests that increased expression, or even dramatically enhanced stability, of ECE1c^WT^ is insufficient to explain the significant elevations in stemness gene expression, self‐renewing capacity, drug resistance, tumorigenesis, and metastatic capability observed both *in vitro* and *in vivo*. However, ET‐1 levels were only quantified after 48 h of growth, which may be too late to observe significant differences between clones. Therefore, differential ET‐1 levels during earlier stages cannot be ruled out, with a potentially significant impact on the CSC traits observed during later stages of growth. Consistent with our results, silencing of ECE1 in ovarian cancer cells has been shown to decrease ET‐1 production, MMP‐2 activity, and invasiveness. Likewise, ET‐1 supplementation reverted the silencing effect, suggesting that ECE1 may exert its role through continuous production of ET‐1 (Rayhman *et al.*, 2008). By contrast, while ECE1c silencing decreased the invasiveness of prostate cancer cells, ET‐1 supplementation did not restore the level of invasiveness (Lambert *et al.*, 2008). Additionally, similar decreases in invasiveness were observed in DLD‐1 and HT‐29 colorectal cancer cells after application of the ECE1 inhibitor SM19712 and siRNA silencing of ECE1c (Niechi *et al.*, 2015). These data strongly suggest a putative ET‐1‐independent mechanism for ECE1c‐enhanced aggressiveness which, interestingly, seems to occur not only in colorectal cancers cells but in other cancer models as well.

Super‐stability of ECE1c led to several CSC‐linked traits in our CRC cells, including self‐renewal and chemoresistance *in vitro*, as well as tumorigenesis *in vitro* and *in vivo*. These findings are consistent with loss of polarity and EMT, that is, acquisition of the mesenchymal phenotype necessary to invade the extracellular matrix and migrate to distant sites, which is a known capacity of CSCs (Polyak and Weinberg, 2009). In fact, typical EMT markers such as Snail, N‐cadherin, and E‐cadherin were also modulated in our work. In addition, ET‐1 supplementation has been observed to increase Snail and N‐cadherin while decreasing E‐cadherin mRNA levels in colorectal cancer cells, while the opposite effects are observed with an ET_A_R receptor antagonist (Cianfrocca *et al.*, 2017). These data are consistent with other studies in which ET‐1 and ET_A_R receptor expression were necessary for lung colonization (Said *et al.*, 2011). Likewise, ET_A_R overexpression promoted hepatic colonization in a colorectal cancer metastasis model (Nie *et al.*, 2014). Moreover, acquired invasive capacity is known to enable colonization of distant tissues and generation of metastatic foci (Shiozawa *et al.*, 2013). Indeed, mice intravenously injected with our CS^K6R^ cells generated a dramatically large number of metastatic lung nodules. In addition, our aggressive CS^K6R^ cells led to significantly decreased survival in the mice, with some animals showing livers with a marked venous thrombosis but, more importantly, all showing massive lymphangitic carcinomatosis in the lungs. Indeed, this clinical finding is often secondary to adenocarcinoma in patients, occurring when the tumor spreads through the lymph system, leading to rapid progression and deterioration of the patient. Thus, lymphangitic carcinomatosis is a marker of poor prognosis, with nearly half of patients expiring within 1 year of this finding (Biswas and Sriram, 2015).

The fact that expression of a super‐stable ECE1c^K6R^ can lead to emergence of CSC‐like traits in CRC cells is, to our knowledge, a novel and highly relevant finding. The evidence presented here suggests the existence of an epigenetic mechanism of enhanced aggressiveness of CRC cells, which involves CK2‐dependent phosphorylation of ECE1c. Thus, a tumor core could be envisaged harboring at least three types of cells: (a) cells with normal ECE1c levels but CK2 aberrantly increased (amount and/or activity), thereby improving ECE1c stability by phosphorylation; (b) cells with normal CK2 levels but ECE1c increased due to mutations at K6, S18, and/or S20; and (c) cells with normal CK2 and ECE1c levels but aberrant expression and/or function of a hypothetical factor linking CK2‐phosphorylation and blockage of proteasomal degradation. Perhaps the most probable scenario is the first, as no mutations at K6, S18, or S20 have been found (J.C. Tapia, personal communication) nor has a proteasome‐blocking factor been reported. Whatever the cell type, our findings predict that in the presence of augmented ECE1c levels, CSCs traits will appear and the tumor will become more aggressive, leading to greater recurrence and/or metastasis. Thus, ECE1c could contribute to cancer aggressiveness in an epigenetic manner as we have recently suggested (Tapia and Niechi, 2019). Therefore, combining specific CK2 and ECE1c inhibitors can be envisioned as a potential therapy for this devastating disease.

## 5 Conclusions

Protein stability of ECE1c is dramatically improved by switching lysine 6 to arginine. This augmented stability is independent on the treatment of CRC cells with the CK2 inhibitor silmitasertib. Overexpression of a super‐stable ECE1c^K6R^ mutant promotes generation of stem‐like population in CRC cells. These cells show increased *in vitro* CSC‐related traits, including expression of stemness genes, spheroid‐formation capacity, 5‐FU resistance, clonogenesis, and invasiveness, as well as *in vivo* traits such as tumor growth and metastasis capacities. These findings suggest that ECE1c could be a new therapeutic target to prevent colorectal cancer aggressiveness in patients.

## Conflict of interest

The authors declare no conflict of interest.

## Author contributions

PP‐M and JCT wrote the manuscript, which was reviewed by LJ and VAB. MV‐G performed lentiviral transduction and cell cloning. SI performed IHCs. IN, HH, PC, FA, and VAB performed MTS, western blots, RT‐qPCR, migration, invasion, clonogenesis, and spheroidogenesis. PP‐M and HH performed statistical analysis, side population, and *in vivo* assays.

## Supporting information


**Fig. S1.** Structural features of ECE1’s N‐terminus end. (A) Primary sequence alignment of the N‐terminus end of the four known human ECE1 isoforms. (B) Alignment of the N‐terminus end of ECE1c isoforms from several mammalian species. Here is shown in bold the conserved sites for putative ubiquitination, Lys‐6, and phosphorylation for protein kinase CK2, Ser‐18 and Ser‐20. Modified from Tapia & Niechi, 2019.

**Fig. S2.** ECE1c^K6R^ promotes expression of stemness genes in CRC cells. Stable clones expressing either Flag‐tagged ECE1c^WT^ or ECE1c^K6R^ generated in HT‐29 (A) and SW‐480 (B) cells were grown under normal conditions for 48 h, and the mRNA levels of the indicated stemness genes were quantified using RT‐qPCR. Data represent average ± SEM (n=3). Anova plus Tukey tests were used. *p≤0.05, **p≤0.01.
