# Supplementary figures and images for: Endothelin‐converting enzyme‐1c promotes stem cell traits and aggressiveness in colorectal cancer cells
Source: Mol Oncol. 2019 Dec 19;14(2):347–62. doi: 10.1002/1878-0261.12609 (PMC6998658; doi:10.1002/1878-0261.12609)

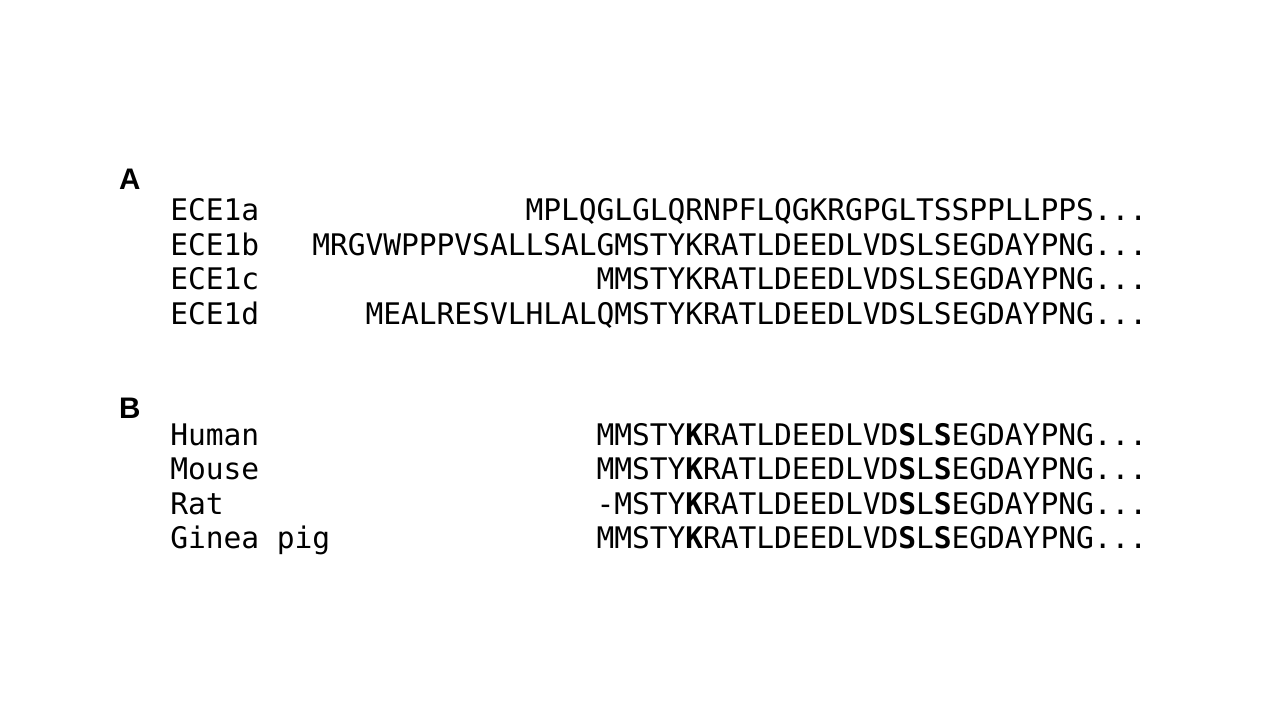

Supplement: Supplementary file 1 — Fig. S1. Structural features of ECE1’s N‐terminus end. (A) Primary sequence alignment of the N‐terminus end of the four known human ECE1 isoforms. (B) Alignment of the N‐terminus end of ECE1c isoforms from several mammalian species. Here is shown in bold the conserved sites for putative ubiquitination, Lys‐6, and phosphorylation for protein kinase CK2, Ser‐18 and Ser‐20. Modified from Tapia & Niechi, 2019. [file MOL2-14-347-s001.tif]

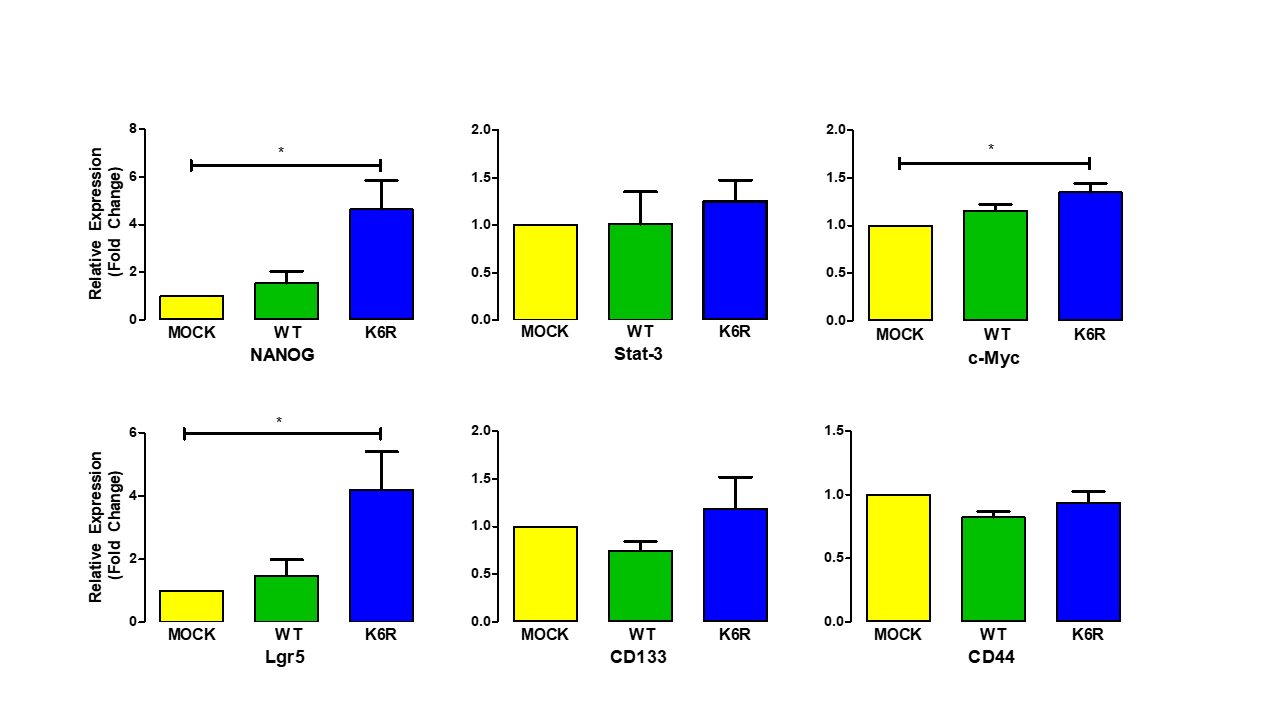

Supplement: Supplementary file 2 — Fig. S2. ECE1cK6R promotes expression of stemness genes in CRC cells. Stable clones expressing either Flag‐tagged ECE1cWT or ECE1cK6R generated in HT‐29 (A) and SW‐480 (B) cells were grown under normal conditions for 48 h, and the mRNA levels of the indicated stemness genes were quantified using RT‐qPCR. Data represent average ± SEM (n=3). Anova plus Tukey tests were used. *p≤0.05, **p≤0.01. [file MOL2-14-347-s002.tif]

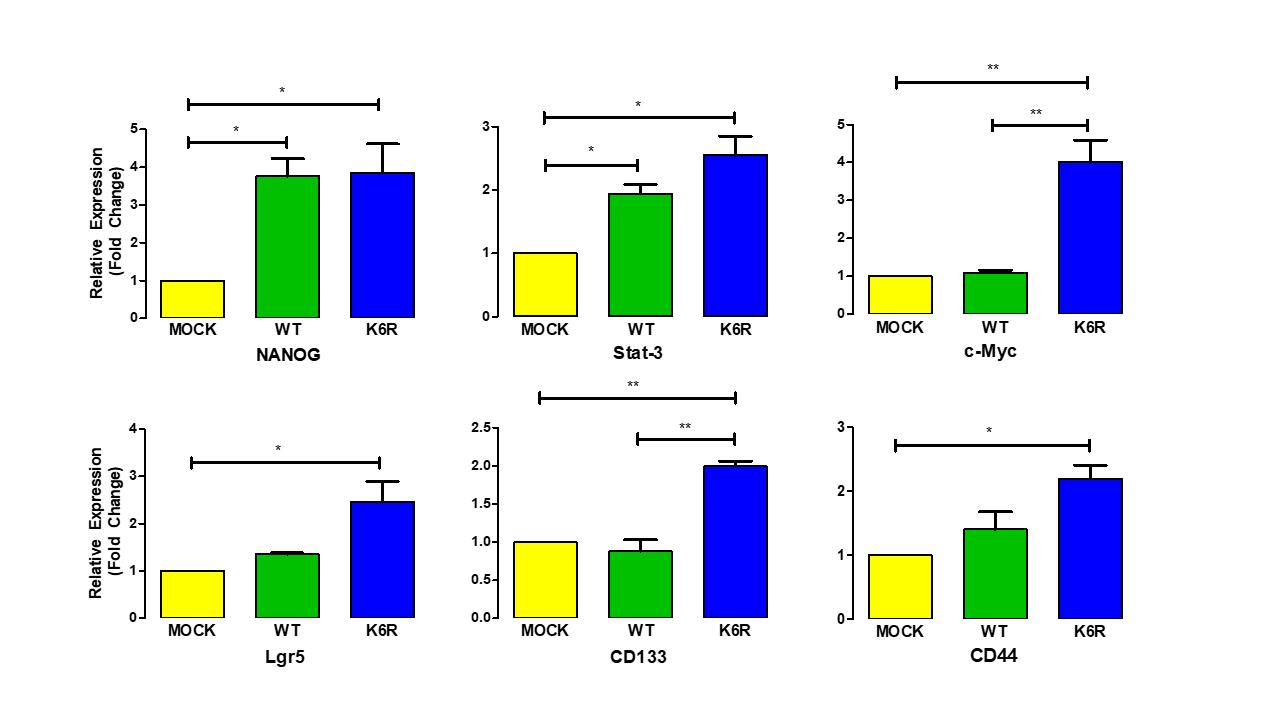

Supplement: Supplementary file 3 [file MOL2-14-347-s003.tif]
